# Impacts of enzymes and probiotic in improving the utilization of sieved olive pulp meal in growing rabbit diets

**DOI:** 10.5455/javar.2024.k761

**Published:** 2024-03-31

**Authors:** Abd-Alfattah A. Alderey, Nabila E.M. El-Kassas, Eman A. Hussein, Soha A. Farag, Ayman A. Hassan, Safaa E.S. Atia, Manal H.A. Gomaa, Eman S. El-Hadad, Salma H. Abu Hafsa

**Affiliations:** 1 Animal Production Research Institute, Agricultural Research Center, Ministry of Agriculture, Dokki, Giza, Egypt; 2 Department of Poultry and Fish Production, Faculty of Agriculture, University of Menoufia, Shibin El-Kom, Egypt; 3 Department of Animal Production, Faculty of Agriculture, Tanta University, Tanta, Egypt; 4 Livestock Research Department, Arid Lands Cultivation Research Institute, City of Scientific Research and Technological Applications, New Borg El-Arab, Alexandria, Egypt

**Keywords:** Olive pulp, Enzyme, Yeast, Performance, Antioxidants status, Rabbits

## Abstract

**Objective:**

This research assesses the utilization of sieved olive pulp (SOP) in the diet of growing rabbits through the use of an exogenous enzyme or dry yeast as a pretreatment.

**Materials and Methods:**

One hundred sixty-eight male V-Line rabbits aged 5 weeks (weighing 550 ± 25 gm) were randomly divided into seven groups with eight replicates each as follows: the control group was fed a basal diet without supplementation; while the other six groups were fed basal diets containing 20% and 25% of SOP and supplemented with 0.1 gm/kg Econase (E), 0.5 gm/kg dry yeast (Y), and a combination of both. The experiment lasted for 8 weeks.

**Results:**

The results indicated that supplementation of E, Y, and EY into rabbit diets containing SOP improved live body weight, body weight gain, feed conversion ratio, and nutrient digestibility. A higher dressing percentage was observed in the groups fed a 20% and 25% SOP diet supplemented with EY (*p* < 0.05). The treated groups showed an increase in total protein, albumin, globulin, A/G ratio, total antioxidant capacity, superoxide dismutase, and glutathione peroxidase (*p* < 0.05), while there was a significant decrease in triglycerides, total cholesterol, and malondialdehyde levels *(p* < 0.05) compared to the control. Rabbit groups fed an SOP diet supplemented with E, Y, or EY demonstrated higher (*p* < 0.05) economic efficiency compared to the control.

**Conclusion:**

Supplementing the diet of rabbits containing SOP with exogenous enzymes and/or dry yeast enhances the nutritional value of SOP while improving rabbit performance, nutrient digestibility, and antioxidant status.

## Introduction

Currently, the exploitation and valorization of agro-food residues and by-products is gaining increased attention. Simultaneously, the expanding need for the utilization of by-products for rabbit farming can be justified by the growing demand in global markets, especially in developing countries like Egypt. Olive pulp (OP) is one of the most interesting by-products of olive trees in the Mediterranean region, and it has several bioactive substances with antioxidant, antibacterial, and antifungal characteristics [1]. On the other hand, the use of by-products from different vegetal origins for feed supplementation is gaining popularity due to its eco-friendly use, efficacy, and sustainability, other than avoiding the necessity to eliminate potentially beneficial and valuable by-products of the agro-food system [1].

Nonetheless, attention should be paid to potential secondary metabolites found in olives [2]. Numerous studies have demonstrated that olive oil industry waste can be used as a low-cost alternative feed for animals, improving productivity and animal health, and increasing the income of livestock breeders [3]. However, their high content of lignocellulose makes them resistant to rumen microbial enzymes, and their high content of polyphenols and tannins inhibits the growth of microorganisms in the rumen, which prevents a large portion of their nutrients from being degraded in the rumen and a large portion of their energy from being metabolized [2]. Therefore, its application in animal diets is still relatively limited, primarily reserved for non-intensive animal diets with low productivity and mainly during periods of scarcely available feed resources [3].

Recent worldwide crises, including climate change, the COVID-19 pandemic, and the war in Russia and Ukraine, have made feed resources scarce, and driven up their price exponentially [4]. Therefore, the valorization of agro-industrial by-products, such as those from the extraction of olive oil, as an alternative feed and the improvement of their nutritional value have become an interesting research direction that requires further investigation [1]. Several feeding practices have been established to increase the utilization of OP to produce economically valuable by-products while lowering the environmental pollution load [5]. Previous studies have proved that a portion of the cell-wall polysaccharides of *Trichoderma longibrachiatum* are bio-converted into simple sugars during the pretreatment of solid waste from the olive oil industry with exogenous enzymes produced from this organism. Consequently, it improves the palatability, nutrient digestibility, ruminal fermentation, and growth performance of animals [6]. It has also been demonstrated that exogenous enzymes at feeding time improve nutrient digestion and antioxidant status and reduce blood lipids [7], improving the protein efficiency ratio while lowering the daily caloric conversion ratio in growing rabbits [8].

In addition, recent research has demonstrated that it is beneficial for animals’ diets to incorporate live yeasts, such as *Saccharomyces cerevisiae*, as supplements. *Saccharomyces cerevisiae* produces a variety of co-factors such as peptides, organic acids, and vitamins, which stimulate and facilitate the growth and proliferation of the beneficial rumen microbiota [9–12], improving feed palatability, nutrient digestibility, growth performance, animal health, and offering a clear economic advantage [10,11]. To the best of our knowledge, there is scarce information on the pre-treatment of OP by DY and EE in rabbit feeding. Therefore, this study aims to bio-convert this by-product into enriched alternative feed for rabbits using EE and DY pre-treatments to lessen the environmental pollution resulting from olive oil extraction.

## Materials and Methods

### Ethical approval

The present study was performed at El-Gimmizah Research Station (El-Gharbia governorate), Animal Production Research Institute, Agriculture Research Center, Egypt. All experimental procedures were performed according to the guidelines for experimental animals, and this study was approved and permitted by the Institutional Animal Care and Use Committees of the City of Scientific Research and Technological Applications of the City of Scientific Research and Technological Applications, Alexandria, Egypt, under permit No. 60-3C-8023.

### Collection and preparation of sieved OP (SOP)

Samples of OP were gathered during the olive pressing season from a local semi-automatic olive pressing factory and then transported to the rabbit research farm. The OP was spread on a plastic sheath for sun-drying. The OP was mixed frequently to ensure that the material dried effectively and was covered overnight to prevent moisture. A few days later, the seeds began to separate when the OP was air-dried. In this technique, the majority of the seeds were removed using a 2-mm sieve. The SOP was kept for subsequent use in tight plastic bags. The chemical composition of the SOP sample is illustrated in Table 1.

### Animals, experimental design, and diet

One hundred sixty-eight, 5-week-old male V-Line rabbits, weighing 550 ± 25 gm were randomly distributed to seven groups with eight replicates (three rabbits each). The dietary groups were as follows: control fed a basal diet without supplementation; and the other six groups in which the basal diet was replaced by two levels (20% and 25%) of SOP supplemented with Econase (E) at 0.1 gm, dry yeast at 0.5 gm/kg (Y), or their combination (EY). The exogenous enzyme, endo-1,4-β-xylanase, was a commercial product (ECONASE^®^ XT), while dry yeast (*S. cerevisiae*) was purchased from a commercial company (Alzahraa, Alexandria, Egypt), which contains 8,000,000,000 CFU active/gm. The proximate analysis of SOP was done according to the standard method of the AOAC [13] (Tables 1). The experimental diets were formulated to meet the nutritional requirements of growing rabbits according to the recommendations of de Blas and Mateos [14], as shown in Table 2. Clean, fresh water and a pelleted diet were offered *ad libitum*. The rabbits were housed in galvanized batteries under uniform management conditions in a well-ventilated room, and the temperature during the experiment was 23°C ± 2°C and the humidity was 55%–65%. The experiment was conducted for 8 weeks from January to February 2021.

**Table 1.** The chemical composition of SOP (% as DM basis).

Items	SOP
DM	91.42
OM	88.70
CP	9.77
CF	31.50
EE	12.50
NFE	34.93
Ash	11.30
NDF	49.62
ADF	38.16
DE* (Kcal/kg diet)	1930

DE*, digestible energy was calculated according to Cheeke (1987). DM, dry matter; SOP, sieved olive pulp.

**Table 2.** Ingredients and chemical composition of the experimental diets (% as DM basis).

Ingredients (%)	Control	Treatments					
		SOPE20	SOPY20	SOPEY20	SOPE25	SOPY25	SOPEY25
Sieved olive pulp	00.00	20.00	20.00	20.00	25.00	25.00	25.00
Yellow corn	25.00	16.00	16.00	16.00	15.70	15.70	15.70
Soybean meal (44 % CP)	12.00	11.00	11.00	11.00	10.50	10.50	10.50
Barley	10.00	7.50	7.50	7.50	5.00	5.00	5.00
Wheat bran	20.00	20.00	20.00	20.00	20.00	20.00	20.00
Alfalfa hay	10.00	10.00	10.00	10.00	10.00	10.00	10.00
Sunflower meal	10.00	10.00	10.00	10.00	10.00	10.00	10.00
Clover straw	7.20	1.70	1.70	1.70	0.00	00.00	00.00
Limestone ground	2.00	0.00	0.00	0.00	0.00	0.00	0.00
Di-calcium phosphate	01.00	1.00	1.00	1.00	1.00	1.00	1.00
Molasses	2.00	2.00	2.00	2.00	2.00	2.00	2.00
Vit. and min. premix*	00.30	0.30	0.30	0.30	0.30	0.30	0.30
Common Salt	00.50	0.50	0.50	0.50	0.50	0.50	0.50
Total	100	100	100	100	100	100	100
*Calculated analysis*							
DE kcal/kg	2522	2506	2506	2506	2501	2501	2501
CP%	16.08	16.33	16.33	16.33	16.24	16.24	16.24
EE%	2.63	4.61	4.61	4.61	5.22	5.22	5.22
CF%	12.15	15.81	15.81	15.81	16.58	16.58	16.58
Calcium %	1.02	1.99	1.99	1.99	2.39	2.39	2.39
Available phosphorus %	0.46	0.36	0.36	0.36	0.36	0.36	0.36

*Vitamin and Mineral premix supplied per kg of die: Vitamin A 10,000 IU, Vitamin D3,1,800 UI; Vitamin E, 15 mg; Vitamin K3, 4.5 mg; Vitamin B1, 0.5 mg; Vitamin B2, 4 mg; Vitamin B12, 0.001 mg; Folic acid, 0.1 mg; Pantothenic acid, 7 mg; Nicotinic acid, 20 mg; I, 1 mg; Mn, 60 mg; Cu, 5.5 mg, Zn, 75 mg; Fe, 40 mg; Co, 0.3 mg; Se, 0.08 mg; Robenidine, 52.8 mg, Antioxidant, 0.250 mg. DM, dry matter basis; SOPE20 and SOPE25, fed diets with 20% and 25% SOP supplemented with 0.1% Econase enzyme; SOPY20 and SOPY25, fed diets containing 20% and 25% SOP with dry yeast at 0.5 gm/kg diet; SOPEY20 and SOPEY25, fed diets containing 20% and 25% SOP with a combination of 0.1% Econase enzyme and dry yeast at 0.5 gm/kg diet.

### Growth performance

The final body weight (FBW) of individual rabbits and feed intake (FI; gram/rabbit) were determined weekly throughout the experiment period. Body weight gain (BWG) and feed conversion ratio (FCR; gm feed/gm gain) were calculated. Performance index percentage (PI%) was estimated as follows: PI% = [LBW (Kg) /FCR] ×100.

### Nutrient digestibility

Eight rabbits from each group were housed individually in metabolic cages and kept to adapt for 7 days before the collection period. Feces were collected for five consecutive days (the collection period) before feeding in the morning. The fecal samples were oven-dried at 70°C for 48 h, grounded, and stored at −20°C until chemical analyses. Samples of SOP, feed, and dried feces were analyzed for dry matter (DM), crude protein (CP), crude fiber (CF), ash, and ether extract (EE) according to agent (40°C–60°C) [13]. Acid detergent fiber (ADF) and neutral detergent fiber (NDF) were calculated following the equations of Cheeke [15] as follows: % ADF = 9.432+(0.912 x CF %) and % NDF = 28.924+(0.657 x CF %). The nitrogen-free extract (NFE) value was calculated by a different method as follows: % NFE = 100 % – (% EE + % CP + % Ash + % CF).

### Carcass characteristics

At week 13 of age, eight rabbits were randomly chosen from each group, individually weighed, and starved for 12 h with the provision of water *ad libitum* for carcass evaluation. After complete bleeding, the pelt, viscera, and tail were removed, and the carcass and its components were weighed as total edible parts. The edible parts (liver, heart, and kidneys) were weighed as a percentage of live body weight, while the non-edible parts were expressed as a percentage of pre-slaughter weight. The dressing percentage was calculated by dividing the hot-dressed carcass weight by the pre-slaughter weight and expressed as a percentage.

### Blood biochemical and antioxidant status parameters

5 ml of blood samples were collected at slaughter in non-heparinized tubes, centrifuged directly at 3,000×g for 15 min, and serum samples were collected and stored at −20ºC for further biochemical examinations. Total protein, albumin, total cholesterol, triglycerides, and glucose were measured spectrophotometrically using commercial kits (Biodiagnostic Co., Giza, Egypt) according to the manufacturer’s instructions. Globulin (total protein-albumin) and the A/G ratio were calculated. Total antioxidant capacity (TAC), superoxidase dismutase (SOD), catalase (CAT), glutathione peroxidase (GSH), and malondialdehyde (MDA) were measured using commercial kits (Biodiagnostic Company Giza, Egypt) by following the manufacturer’s instructions.

### Economic efficiency

Costs and revenues were calculated based on the prevailing prices in the Egyptian market at the time of the experiment as follows: Economical efficiency = (A−B/B) × 100. Where: A = price of kg gain in Egyptian pounds B = Feed cost/kg gain in Egyptian pounds.

### Statistically analysis

The data obtained from the experiment were statistically examined by analysis of variance using SPSS software’s general linear model (v. 16.0, SPSS Inc., Chicago, IL) according to the following statistical model:

*Y*_ij_ *= μ + T*_i_ *+ ε*_ij_

**w**here *y*_ij_ is the measured value, μ is the overall mean effect, *T*_i_ is the *i*th treatment effect, and *ε*_ij_ is the random error associated with the *j*th rabbit assigned to the *i*th treatment. Significant differences between the means were determined at *p* < 0.05. All results are presented as least-squares means.

## RESULTS

### The chemical composition of SOP

The chemical composition of SOP for DM, OM, CP, CF, EE, NFE, and ash was (91.42%, 88.70%, 9.77%, 31.50%, 12.50%, 34.93%, and 11.30%), respectively (Table 1).

### Growth performance

The effect of feeding growing rabbits with SOP incorporated with or without E, Y, or EY on their growth performance is shown in Table 3. The initial weights of the rabbits did not significantly differ between the experimental groups, which confirmed their random distribution. Final body weight (FBW) and TBWG were improved (*p* < 0.05) in groups fed SOP diets incorporated with E, Y, or EY compared to the control. When compared to the control, supplementation with EY showed the highest TBWG in groups fed SOP20 and SOP25. During the periods (5–9), (9–13), and (5–13) weeks of age, TBWG was shown to be significantly (*p* < 0.05) significantly improved in the groups fed SOP diets supplemented with E, Y, or EY, while FI was not significantly affected by the experimental treatments. During the periods (5–9), (9–13), and (5–13) weeks of age, rabbits given SOP diets supplemented with E, Y, or EY showed the best FCR. Rabbits fed SOP diets supplemented with EY exhibited the best PI, followed by those fed SOP diets supplemented with E or Y.

### Nutrient digestibility

Table 4 showed that the nutrient digestibility of DM, OM, CP, CF, and EE was affected by the inclusion of both levels of SOP. The addition of Econase and yeast considerably improved all nutrient digestibility in the SOP20 and SOP25 groups compared with the control group.

### Carcass traits

The relative weights of the head, liver, heart, kidneys, trunk, forelimb, hindlimb, total edible parts, and total non-edible parts were not affected by the SOP levels of diets with or without E or Y (Table 5). The rabbits fed 20% and 25% SOP diets supplemented with EY showed the best dressing percentage, followed by the rabbits fed 20% and 25% SOP diets with E and Y when compared with the other groups.

### Blood biochemical and antioxidant status parameters

The concentrations of serum total protein, albumin, and globulin increased significantly (*p* < 0.05), while A/G ratio, total cholesterol, and triglycerides concentrations decreased dramatically (*p* < 0.05) in groups fed SOP diets supplemented with E, Y, or EY (Table 6). Total cholesterol was decreased by 7.7%, 10.8%, 12.6%, 14%, 19.4%, and 18%when growing rabbits were fed 20% and 25% SOP diets supplemented with E, Y, or EY, while triglycerides were decreased by 12.3%, 11.1%, 8.8%, 13.2%, 10.9%, and 12.5%, respectively compared to those fed the control. There were no appreciable differences in the blood glucose levels between the treatment groups. The TAC, SOD, CAT, and GSH were significantly increased in treated groups, while MDA markedly decreased.

**Table 3.** Effect of dietary level of supplementation of SOP with or without exogenous enzyme or dry yeast on growth performance of growing rabbits.

Items	Control	Treatments							SEM	*p*-value
		SOPE20		SOPY20	SOPEY20	SOPE25	SOPY25	SOPEY25		
IBW, g	564.0		574.0	550.0	541.0	534.0	530.0	524.0	17.28	0.845
FBW, g	1856.0^b^		2053.0^a^	2031.0^a^	2089.0^a^	1988.0^a^	2011.0^a^	2081.0^a^	27.06	<0.001
TBWG, g	1292^c^		1469^b^	1481^b^	1548^a^	1454^b^	1481^b^	1557^a^	45.78	0.001
BWG (g/d)										
Weeks 5–9 Weeks 9–13 Weeks 5–13	23.93^b^ 22.21^b^ 23.07^c^		26.11^a^ 24.29^a^ 26.23^ab^	26.75^a^ 25.43^a^ 26.45^ab^	28.06^a^ 25.88^a^ 27.64^a^	25.39^a^ 24.07^a^ 25.94^b^	26.14^a^ 24.75^a^ 26.45^ab^	28.00^a^ 25.03^a^ 27.80^a^	1.27 1.13 0.82	<0.001 0.003 0.005
Feed intake (g/h/d)										
Weeks 5–9 Weeks 9–13 Weeks 5–13	78.80 99.80 89.3		81.00 101.00 90.0	79.10 98.30 89.20	83.20 100.20 91.20	81.50 100.10 90.80	80.00 101.20 90.10	82.90 102.10 91.50	2.41 2.15 2.61	0.792 0.851 0.894
FCR										
Weeks 5–9 Weeks 9–13 Weeks 5–13	3.29^a^ 4.49^a^ 3.87^a^		3.10^ab^ 4.16^b^ 3.43^b^	2.96^b^ 3.87^c^ 3.37^b^	2.97^b^ 3.87^c^ 3.30^b^	3.21^ab^ 4.16^b^ 3.50^b^	3.06^b^ 4.09^b^ 3.41^b^	2.96^b^ 4.08^b^ 3.29^b^	0.15 0.11 0.12	0.019 0.021 0.011
PI, %	47.96^d^		59.85^b^	60.27^b^	63.30^a^	56.80^c^	58.97^b^	63.25^a^	1.21	0.001

^a–d^Means in the same row without similar superscripts are significantly (*p* < 0.05) different. SOPE20 and SOPE25, fed diets with 20% and 25% SOP supplemented with 0.1% Econase enzyme; SOPY20 and SOPY25, fed diets containing 20% and 25% SOP with dry yeast at 0.5 gm/kg diet; SOPEY20 and SOPEY25, fed diets containing 20% and 25% SOP with a combination of 0.1% Econase enzyme and dry yeast at 0.5 gm/kg diet. IBW, initial body weight; FBW, final body weight; TBWG, total BWG; PI, performance index.

**Table 4.** Effect of dietary level of supplementation of SOP with or without exogenous enzyme or dry yeast on growth performance of growing rabbits on nutrient digestibility.

Items	Control	Treatments						SEM	*p*-value
		SOPE20	SOPY20	SOPEY20	SOPE25	SOPY25	SOPEY25		
DM, %	59.02^b^	61.75^a^	61.63^a^	62.42^a^	62.23^a^	61.56^a^	62.36^a^	0.63	0.015
OM, %	60.33^b^	63.25^a^	62.84^a^	63.12^a^	63.72^a^	63.11^a^	63.96^a^	0.74	0.001
CP, %	60.01^b^	62.79^a^	62.52^a^	63.77^a^	62.22^a^	62.72^a^	63.27^a^	0.96	0.007
CF, %	54.31^b^	56.66^a^	55.88^a^	56.30^a^	56.35^a^	55.32^a^	56.36^a^	1.73	0.016
EE, %	77.72^b^	79.02^a^	79.88^a^	79.90^a^	79.26^a^	79.20^a^	80.03^a^	0.88	0.013

^a–b^Means in the same row without similar superscripts are significantly (*p* < 0.05) different. SOPE20 and SOPE25, fed diets with 20% and 25% SOP supplemented with 0.1% Econase enzyme; SOPY20 and SOPY25, fed diets containing 20% and 25% SOP with dry yeast at 0.5 gm/kg diet; SOPEY20 and SOPEY25, fed diets containing 20% and 25% SOP with a combination of 0.1% Econase enzyme and dry yeast at 0.5 gm/kg diet. DM, dry matter; OM, organic matter; CP, crude protein; CF, crude fiber; EE, Ether extract.

### Economic efficiency

Feeding growing rabbits on 20% and 25% SOP diets supplemented with E, Y, or EY reduced the feed cost per kg gain by 19.64%, 21.06%, 21.85%, 21.61%, 23.82% and 25.39%, while net revenue values were increased with 20% and 25% SOP diets supplemented with E, Y, or EY by 28.97%, 30.46%, 37.73%, 29.18%, 33.29% and 41.46%, respectively compared to those fed those fed the control diet (Table 7). In comparison with the control diet. It was clear that the diet containing 25% SOP had the highest economic efficiency and relative economic efficiency followed by the one with 20% SOP.

**Table 5.** Effect of dietary level of supplementation of SOP with or without exogenous enzyme or dry yeast on carcass traits of growing rabbits.

Items	Control	Treatments						SEM	p-value
		SOPE20	SOPY20	SOPEY20	SOPE25	SOPY25	SOPEY25		
Dressing %	61.53^c^	62.66^b^	62.00^ b^	64.16^a^	61.68^b^	61.23^b^	64.20^a^	0.44	0.018
Head %	6.58	6.97	6.91	6.19	7.14	6.40	6.60	0.19	0.883
Liver %	2.81	2.90	2.79	2.63	2.63	3.02	2.76	0.22	0.784
Heart %	0.39	0.36	0.41	0.42	0.40	0.41	0.39	0.04	0.822
Kidney %	0.71	0.69	0.70	0.85	0.71	0.71	0.70	0.06	0.816
Trunk %	12.69	12.75	11.91	12.68	12.44	11.96	12.19	0.61	0.734
fore limb %	17.92	18.83	18.34	16.75	17.75	16.60	16.83	0.81	0.669
Hind limb %	20.33	21.67	20.83	18.11	21.24	20.11	20.17	0.78	0.853
TEP %	61.53	62.16	62.00	60.66	61.36	60.23	61.68	2.13	0.677
TNEP %	38.47	39.84	38.00	40.34	39.64	39.77	38.32	2.13	0.715

^a-c^Means in the same row without similar superscripts are significantly (*p* < 0.05) different. SOPE20 and SOPE25, fed diets with 20% and 25% SOP supplemented with 0.1% Econase enzyme; SOPY20 and SOPY25, fed diets containing 20% and 25% SOP with dry yeast at 0.5 gm/kg diet; SOPEY20 and SOPEY25, fed diets containing 20% and 25% SOP with a combination of 0.1% Econase enzyme and dry yeast at 0.5 gm/kg diet. TEP, total edible parts; TNEP, total non-edible parts.

**Table 6.** Effect of dietary level of supplementation of SOP with or without exogenous enzyme or dry yeast on blood biochemical profile and antioxidant status.

Items		**Treatments**							
	Control	SOPE20	SOPY20	SOPEY20	SOPE25	SOPY25	SOPEY25	SEM	*p*-value
*Serum biochemistry*									
Total protein, g/dl	5.43^b^	6.43^a^	6.37^a^	6.40^a^	6.27^a^	6.16^a^	6.33^a^	0.23	0.002
Albumin, g/dl	3.28^b^	3.64^a^	3.57^a^	3.63^a^	3.52^a^	3.50^a^	3.66^a^	0.15	0.013
Globulin, g/dl	2.15^b^	2.79^a^	2.80^a^	2.77^a^	2.75^a^	2.66^a^	2.67^a^	0.19	0.017
A/G ratio	1.53^a^	1.30^b^	1.28^b^	1.31^b^	1.28^b^	1.32^b^	1.37^b^	0.06	0.011
Cholesterol, mg/dl	74.00^a^	68.33^b^	66.00^b^	64.67^b^	63.67^bc^	59.67^c^	60.67^c^	4.99	0.009
Triglycerides, mg/dl	143.67^a^	126.00^b^	127.67^b^	131.00^b^	124.67^b^	128.00^b^	125.67^b^	5.53	0.556
Glucose, mg/dl	110.00	117.67	110.67	110.67	109.67	101.00	109.67	1.29	0.685
*Antioxidant status*									
TAC, U/L	3.53^d^	5.73^c^	8.47^a^	8.33^a^	5.10^c^	6.30^b^	7.53^a^	0.57	<0.001
SOD, U/L	237.00^b^	351.00^a^	385.67^a^	382.33^a^	341.67^a^	379.00^a^	361.33^a^	53.41	<0.001
CAT, U/L	0.22^b^	0.61^a^	0.74^a^	0.78^a^	0.57^a^	0.69^a^	0.73^a^	0.17	0.011
GSH, U/L	2.53^b^	4.73^a^	5.47^a^	5.87^a^	4.93^a^	5.83^a^	5.97^a^	0.33	<0.001
MDA, U/L	73.00^a^	50.00^b^	39.33^c^	31.33^d^	53.67^b^	38.33^c^	29.33^d^	3.42	<0.001

^a–d^Means within a row with different superscripts are significantly different (*p* < 0.05). SOPE20 and SOPE25, fed diets with 20% and 25% SOP supplemented with 0.1% Econase enzyme; SOPY20 and SOPY25, fed diets containing 20% and 25% SOP with dry yeast at 0.5 gm/kg diet; SOPEY20 and SOPEY25, fed diets containing 20% and 25% SOP with a combination of 0.1% Econase enzyme and dry yeast at 0.5 gm/kg diet. TAC, total antioxidant capacity; SOD, super oxidase dismutase; CAT, catalase; GSH, glutathione perioxidase; MDA, malondialdehyde.

## Discussion

The chemical composition of SOP The content of CP (9.8%) of SOP without seeds was within the normal ranges reported by Abd El-Dayem [16] (8.8%) and Farahat et al. [17] (8.0%); however, it was greater than that reported by Dorbane et al. [18] (6.4%). SOP contains a notable amount of CF (31.5%) that is lower than that reported by Dorbane et al. [18] (45%), but higher than that published by Abd El-Dayem [16] (26.1%) and Farahat et al. [17] (23.8%). In addition, SOP presents a high fat content of 12.5%, which is comparable to that reported by Abd El-Dayem [16] (12%) and Farahat et al. [17] (12.4%). However, it surpasses the 8.2% reported by Dorbane et al. [18]. The resulting 11.3% content of ash was considered high, and it was comparable to that reported by Abd El-Dayem [16] (8.2%) but higher than that reported by Dorbane et al. [18] (2.6%) and Farahat et al. [17] (5.4%). These differences may be due to the type of olive, stage of maturity, climate, manufacturing process, oil extraction degree, year, and geographical origin of the olive, according to De Blas et al. [19]. Animal nutritionists and animal producers are primarily concerned with the availability of low-cost raw ingredients with high nutritional value for animal feed. OP is a by-product of the olive oil industry, which is economically valuable and has high nutritional value [20,21]. The results demonstrated that 20 and 25% SOP with Econase (E) and dry yeast (Y) gave better growth performance, including FBW and TBWG, FCR, and PI for rabbits. The findings of this study showed that adding E or Y to the SOP diet of rabbits enhanced the nutritional value of SOP. These findings revealed that rabbits fed 20% and 25% SOP with E or Y were more efficient in feed utilization than those receiving a control diet. These findings partially agreed with those reported by [21], who found that supplementing the olive cake diet with *S. cerevisiae* improved BWG and enhanced nutrient digestibility. On the other hand, LBW, FI, and FCR did not differ between treatment groups compared to the control. Growing rabbit diets supplemented with *S. cerevisiae* showed better BWG and FCR [10,11]. The addition of probiotic organisms to rabbits’ feeds improved BWG and FCR [22]. In addition, exogenous enzymes enhanced growth performance, protein efficiency ratio, and nutrient digestibility, as shown by Abu Hafsa et al. [7]. The noticeable enhancement in productive performance in our findings suggests that E or Y contributes to the improvement of the intestinal microbial environment by lowering pH, eliminating pathogens, and reducing anti-nutrient levels in SOP, all of which improve rabbit health and feed utilization efficiency [7,10,11,21]. Azazi et al. [23] reported that growing rabbits fed diets with 10% olive cake + 0.1% citric acid had greater PI than the other groups.

**Table 7.** Economic Efficiency of growing rabbits fed dietary level of supplementation of SOP with or without exogenous enzyme or dry yeast.

Items	Control	Treatments					
		SOPE20	SOPY20	SOPEY20	SOPE25	SOPY25	SOPEY25
Total weight gain, kg	1.292	1.469	1.481	1.548	1.454	1.481	1.557
Price of kg gain, LE	50	50	50	50	50	50	50
Revenue LE/rabbit	64.60	73.45	74.05	77.40	72.70	74.05	77.85
Feed intake kg/rabbit	5.00	5.04	5.00	5.11	5.08	5.04	5.12
Price of kg diet, LE	5.25	4.76	4.75	4.81	4.56	4.55	4.61
Total feed cost, LE	26.25	23.99	23.75	24.58	23.16	22.93	23.60
Net revenue LE	38.35	49.46	50.03	52.82	49.54	51.12	54.25
Economic Efficiency	1.46	2.06	2.11	2.15	2.14	2.23	2.30
Relative economic efficiency	100	141.10	144.52	147.26	146.58	152.74	157.53

LE = Egyptian pound. Total feed cost (LE) = (Price of kg feed intake (LE) × Feed intake (kg)/rabbit. Net revenue = BWG revenue - Total feed cost. Economic efficiency = Net revenue/Total feed cost. Relative economic efficiency, assuming the control treatment = 100%. SOPE20 and SOPE25, fed diets with 20% and 25% SOP supplemented with 0.1% Econase enzyme; SOPY20 and SOPY25, fed diets containing 20% and 25% SOP with dry yeast at 0.5 gm/kg diet; SOPEY20 and SOPEY25, fed diets containing 20% and 25% SOP with a combination of 0.1% Econase enzyme and dry yeast at 0.5 gm/kg diet. Feed cost/kg gain for all treatments are as follows: Control = 20.32; SOPE20 = 16.33; SOPY20 = 16.04; SOPEY20 = 15.88; SOPE25 = 15.93; SOPY25 = 15.48; SOPEY25 =15.16, respectively.

Our findings suggest that nutrient digestibility was improved in rabbits fed SOP diets supplemented with Econase and yeast probiotics, which in turn led to better rabbit growth performance. Feeding rabbit diets with up to 20% olive cake resulted in a considerable increase in EE [23]. Mehrez and Mousa [24] found a significant increase in EE when substituting olive cake with barley grains (20%, 25%, or 30%) in the rabbit diet. Econase can enhance the activity of digestive enzymes, resulting in further biodegradation of plant cell walls. In addition, the incorporation of yeast into SOP diets in this study has an impact on the rabbit’s performance in addition to enhancing the nutritional value of the diet.

Econase was added to SOP, which accelerated the biodegradation of CF and caused a further decrease in CF content and an increase in CP content. Yeast has a well-established history of being utilized to enhance diets that include agro-industry by-products [1]. In the literature, yeast has been observed to enhance digestion by encouraging the growth of beneficial organisms and enhancing gut morphology and ecology [12]. This, in turn, improves feed utilization, health status, and immunity in animals [1]. Exogenous enzymes and yeast supplements in rabbit feed might have worked synergistically to improve gut health and ecology, which supported better nitrogen utilization and positively affected growth [10–12].

Different levels of SOP with supplements did not affect the carcass characteristics; adding EY in the 20% and 25% SOP groups increased the dressing percentage. The addition of Econase or dry yeast may have improved dressing weight due to their role in improving nutrient digestibility by modifying gut morphology and gut microbial content (reduction in pathogenic load), leading to improved nutrient digestion and absorption and enhanced FCR [7,9–11]. Consistent with our findings, Mehrez and Mousa [24] found that feeding rabbits olive cake pulp does not affect their carcass traits. The inclusion of exogenous enzymes and yeast in the rabbit diet has been reported to enhance dressing weight in rabbits [7,10,11]. These differences in the impact of probiotics on carcass characteristics might be attributed to several factors, such as diet composition, probiotic dosage and strain, and animal species like age, sex, or breed [25]. The current study found that including different levels of SOP with Econase and dry yeast supplements increased total protein, albumin, and globulin, while decreasing cholesterol and triglyceride levels, except the A/G ratio did not differ among experimental groups. These findings suggest that the experimental diets did not cause any stress or damage to the rabbits’ livers. Our results are partially consistent with studies conducted by Abu Hafsa et al. [7] on exogenous enzymes [7] and [10–12] on yeast, which concluded that incorporating these supplements into rabbit diets improved blood protein, albumin, and globulin levels while lowering cholesterol and triglyceride levels. It is possible that the treatment groups’ reduced cholesterol levels resulted from their ability to incorporate cholesterol into their cellular membrane and convert it into coprostanol, which is directly excreted with the feces, thus reducing blood cholesterol [26].

The results of this investigation corroborated those of Elbaz et al. [21], who reported that feeding OP with probiotics reduced blood cholesterol. The current findings showed the beneficial influence of yeast on serum lipid components, and these observed results are consistent with those of Tollba et al. [27], who demonstrated that the Yea Sacc supplement lowered cholesterol. According to Abdel-Azeem et al. [28], Yea Sacc significantly decreased cholesterol. Yeast (*S. cerevisiae*) decreased serum cholesterol and triglycerides [12,29]. This reduction may be due to the ability of yeasts to absorb or degrade cholesterol and bile salts, followed by conjugation to prevent the resynthesis of cholesterol. Other studies illustrated that the addition of probiotics did not affect cholesterol [30,31]. Because OP contains unsaturated and polyunsaturated fatty acids, research has generally supported that it has advantages in lowering cholesterol levels [32,33]. Blood cholesterol and triglyceride levels decreased when the olive cake was added to the broiler meal [34].

Antioxidant enzymes are not only essential scavengers of free radicals that protect the body tissues from oxidative stress damage [35,36] but also prevent meat from deteriorating [37]. Therefore, enhancing the antioxidant properties of meat is crucial to improving meat quality and extending the shelf life of meat. In the current study, rabbits who received different levels of SOP with Econase or yeast probiotics were shown to have the highest serum antioxidant capacity. The addition of SOP results in some phenolic compounds (hydroxytyrosol, glycoside, and oleuropein), which function as potential antioxidants and cause a discernible improvement in oxidation. This, in turn, strengthens the animals’ defenses against disease [21]. In addition, exogenous enzymes promote antioxidants that can protect cells from free radicals, reduce toxicity, and potentially protect the liver [7]; and yeast probiotics generate a range of substances that suppress cytotoxicity, scavenge free radicals, and capture reactive oxygen species [38].

According to Ibrahim et al. [39], the groups that were fed olive pomace subjected to microbial and enzymatic fermentation exhibited the highest serum antioxidant capacity. Administration of olive by-products to rabbits’s diets improved oxidative stability [33]. Similar results were observed in rabbit groups given different doses of SOP in their diet, leading to improved SOD activity, according to Oke et al. [40]. In addition, Abu Hafsa et al. [7] reported that the antioxidative status in rabbits can be improved by adding exogenous enzymes to their diets, which enhance their antioxidant properties by increasing TAC, superoxide dismutase, and CAT and decreasing MDA levels. The results of [9–11] showed that the yeast supplement improved antioxidant properties, which strengthened the animals’ defenses against disease [9–11].

The productivity of a flock can be determined by an economic production evaluation. Feed is a significant input that contributes ~70%–80% of the total cost of rearing rabbits. In addition, employing feed additives such as Econase and yeast probiotics is an affordable technique to enhance the nutritional value of novel non-conventional feed ingredients such as olive by-products and improve the growth performance and immune function of rabbits. Improving the efficiency of low-quality products and reducing nutrient loss through the utilization of Econase and yeast probiotics might have a substantial impact on the productivity of poultry and animals, with potential economic benefits. According to the present study, the incremental expenses of adding Econase and dry yeast to SOP were low compared to the cost of conventional feed ingredients. The use of 20% SOP followed by 25% SOP with E, Y, or EY was more economically profitable than the control diet since these combinations offered better growth performance and profit-earning. An economic evaluation of adding up to 25% of OP combined with Econase and yeast to rabbit diets revealed that the resulting cost and profit indices were superior to those of diets based on corn [32].

## Conclusion

The present study concluded that adding exogenous enzymes and/or dry yeast to the diet of rabbits containing SOP improves the nutritional value of SOP without negatively affecting the growing rabbits’ nutritional and physiological profiles, which in turn enhances rabbits’ performance, nutrient digestibility, and antioxidant status. These findings further suggest that, when combined with exogenous enzymes and/or dried yeast, SOP can be utilized in rabbit diets at a rate of up to 25%, offering an avenue to compensate for the feedstuff shortage in the rabbit sector.

## List of Abbreviations

ADF, acid detergent fiber; CAT, catalase; CF, crude fiber; CP, crude protein; DM, dry matter; DE, digestible energy; E, Econase enzyme; EE, ether extract; FBW, final body weight; FI, feed intake; FCR, feed conversion ratio; GSH, glutathione peroxidase; IBW, initial body weight; LE, Egyptian pound; MDA, malondialdehyde; NDF, neutral detergent fiber; NFE, nitrogen free extract; Expansion of the Abbreviation “NRC” is deleted from the “List of Abbreviations” section, because it is replaced with de Blas and Mateos (2020). OM, organic matter; PI, Performance index; *p*-value, probability value; SOP, sieved olive pulp; SOD, super oxidase dismutase; SPSS, Statistical Product and Service Solutions; TBWG, total body weight gain; TEP, total edible parts; TNEP, total non-edible parts; TAC, total antioxidant capacity; Y, dry yeast.
